# Correction for Vangay et al., “Microbiome Metadata Standards: Report of the National Microbiome Data Collaborative’s Workshop and Follow-On Activities”

**DOI:** 10.1128/mSystems.00273-21

**Published:** 2021-05-04

**Authors:** Pajau Vangay, Josephine Burgin, Anjanette Johnston, Kristen L. Beck, Daniel C. Berrios, Kai Blumberg, Shane Canon, Patrick Chain, John-Marc Chandonia, Danielle Christianson, Sylvain V. Costes, Joan Damerow, William D. Duncan, Jose Pablo Dundore-Arias, Kjiersten Fagnan, Jonathan M. Galazka, Sean M. Gibbons, David Hays, Judson Hervey, Bin Hu, Bonnie L. Hurwitz, Pankaj Jaiswal, Marcin P. Joachimiak, Linda Kinkel, Joshua Ladau, Stanton L. Martin, Lee Ann McCue, Kayd Miller, Nigel Mouncey, Chris Mungall, Evangelos Pafilis, T. B. K. Reddy, Lorna Richardson, Simon Roux, Lynn M. Schriml, Justin P. Shaffer, Jagadish Chandrabose Sundaramurthi, Luke R. Thompson, Ruth E. Timme, Jie Zheng, Elisha M. Wood-Charlson, Emiley A. Eloe-Fadrosh

**Affiliations:** a Lawrence Berkeley National Laboratory, Berkeley, California, USA; b European Molecular Biology Laboratory, European Bioinformatics Institute, Wellcome Genome Campus, Hinxton, Cambridge, UK; c National Center for Biotechnology Information, National Library of Medicine, National Institutes of Health, Bethesda, Maryland, USA; d IBM Almaden Research Center, San Jose, California, USA; e NASA Ames Research Center, Moffett Field, California, USA; f Biosystems Engineering Department, University of Arizona, Tucson, Arizona, USA; g Los Alamos National Laboratory, Los Alamos, New Mexico, USA; h California State University, Monterey Bay, California, USA; i Institute for Systems Biology, Seattle, Washington, USA; j Department of Bioengineering, University of Washington, Seattle, Washington, USA; k Center for Bio/Molecular Science & Engineering, Naval Research Laboratory, Washington, DC, USA; l Department of Botany and Plant Pathology, Oregon State University, Corvallis, Oregon, USA; m Department of Plant Pathology, University of Minnesota, St. Paul, Minnesota, USA; n Oak Ridge National Laboratory, Oak Ridge, Tennessee, USA; o Pacific Northwest National Laboratory, Richland, Washington, USA; p Institute of Marine Biology Biotechnology and Aquaculture, Hellenic Centre for Marine Research, Heraklion, Crete, Greece; q Department of Energy Joint Genome Institute, Berkeley, California, USA; r Department of Pediatrics, School of Medicine, University of California, San Diego, California, USA; s Northern Gulf Institute, Mississippi State University, Mississippi State, Mississippi, USA; t Ocean Chemistry and Ecosystems Division, Atlantic Oceanographic and Meteorological Laboratory, National Oceanic and Atmospheric Administration, Miami, Florida, USA; u US Food and Drug Administration, Center for Food Safety and Applied Nutrition, College Park, Maryland, USA; v Department of Genetics, Perelman School of Medicine, University of Pennsylvania, Philadelphia, Pennsylvania, USA; w University of Maryland School of Medicine, Institute for Genome Sciences, Baltimore, Maryland, USA

## AUTHOR CORRECTION

Volume 6, no. 1, e01194-20, 2021, https://doi.org/10.1128/mSystems.01194-20. The article byline and affiliation line should read as given in this correction.

